# Poisonous Plant Prediction Using Explainable Deep Inherent Learning Model

**DOI:** 10.3390/s25144298

**Published:** 2025-07-10

**Authors:** Ahmed S. Maklad, Ashraf Alyanbaawi, Mohammed Farsi, Hani M. Ibrahim, Mahmoud Elmezain

**Affiliations:** 1 College of Computer Science and Engineering, Taibah University, Yanbu 966144, Saudi Arabia; ayanbaawi@taibahu.edu.sa (A.A.); mafarsi@taibahu.edu.sa (M.F.); habdelrhman@taibahu.edu.sa (H.M.I.); mmahmoudelmezain@taibahu.edu.sa (M.E.); 2 Information Systems Department, Faculty of Computers and Artificial Intelligence, Beni-Suef University, Beni-Suif 62521, Egypt; 3 Mathematics & Computer Science Department, Faculty of Science, Menoufiya University, Menoufia 32511, Egypt; 4 Computer Science Department, Faculty of Science, Tanta University, Tanta 31527, Egypt

**Keywords:** deep inherent learning, XAI, image classification, interpretability, explanation techniques

## Abstract

The increasing global discovery of plant species presents both opportunities and challenges, particularly in distinguishing between beneficial and poisonous varieties. While computer vision techniques show promise for classifying plant species and predicting toxicity, the lack of comprehensive datasets including images, scientific names, descriptions, local names, and poisonous status complicates these predictions. In this paper, we propose an Explainable Deep Inherent Learning approach that leverages advanced computer vision techniques for effective plant species classification and poisonous status prediction. The proposed Deep Inherent Learning method was validated using different explanation techniques, and Explainable AI (XAI) was employed to clarify decision-making processes at both the local and global levels. Additionally, we provide visual information to enhance trust in the proposed method. To validate the efficacy of our approach, we present a case study involving 2500 images of 50 different plant species from the Arabian Peninsula, enriched with essential metadata. This research aims to reduce the incidence of poisoning from harmful plants, thereby benefiting individuals and society. Our experimental results demonstrate strong performance, with the XAI model achieving accuracy, Precision, Recall, and F1-Score of 0.94, 0.96, 0.96 and 0.97, respectively. By enhancing interpretability, our study fosters greater trust in AI-driven plant classification systems.

## 1. Introduction

Plants play a vital role in agriculture, biodiversity, and environmental sustainability. However, plant diseases and toxic species continue to pose serious threats to food security, public health, and the ecological balance. Leaf diseases, in particular, are among the most destructive issues in plant health, significantly reducing crop yields and quality [1,2]. Globally, approximately 37% of crop yield losses are attributed to invasive plants and weed-related challenges [3], highlighting the need for intelligent and automated plant health monitoring systems.

While the expanding discovery of plant species supports advancements in medicine, agriculture, and conservation, it also increases the complexity of distinguishing between edible, medicinal, and toxic plants [4,5]. In many regions, especially those dependent on traditional or local knowledge, the misidentification of poisonous plants can lead to serious health consequences or even fatalities [6]. Therefore, the accurate and interpretable classification of toxic plant species is a critical task in both the agricultural and public health domains.

Recent advancements in deep learning (DL), particularly through convolutional neural networks (CNNs), have greatly improved image-based plant classification and disease detection [7,8]. These models offer high accuracy and are widely used in smart agriculture applications [9,10]. However, existing studies have largely focused on disease detection and general plant species recognition, with limited attention paid to the classification of poisonous plants.

Moreover, two critical gaps hinder further progress in this area: First, there is a lack of high-quality, structured datasets that combine leaf images with comprehensive botanical metadata such as scientific names, local names, toxicity status, and morphological descriptions [11,12]. Second, most DL models operate as black boxes, offering limited interpretability. This lack of transparency diminishes user trust, especially in high-stakes domains like toxicology and environmental safety [13]. To address these challenges, this research introduces a novel Explainable Deep Inherent Learning (EDIL) model to classify poisonous and non-poisonous plants using leaf images. The proposed approach is robust, interpretable, and deployable in real-world agricultural and ecological environments.

### Main Contributions

The major contributions of this study are summarized as follows:

- A novel EDIL model is proposed for classifying poisonous and non-poisonous plant species based on leaf images.
- A multi-class classification framework is developed and trained on a curated dataset comprising 2500 images from 50 Arabic plant species, enriched with scientific names, local names, toxicity status, and botanical descriptions.
- A Latent Diffusion Model (LDM) is used for data augmentation, increasing the dataset to 7500 images and enhancing model generalization across diverse conditions.
- The model incorporates Explainable AI (XAI) techniques such as occlusion sensitivity and convolutional feature visualization to generate visual justifications for predictions and to improve user trust.
- An ensemble of pretrained CNN architectures is employed to increase accuracy, robustness, and adaptability across varied plant species and environmental settings.
- The proposed system achieves promising classification results, with 94% accuracy, 96% Precision and Recall, and a 97% F1-score, indicating its potential effectiveness for poisonous plant identification.
- The model is designed with computational efficiency in mind, making it potentially suitable for deployment on edge devices to support practical toxic plant identification in field settings.
- This work bridges the gap between high-performance DL and transparent, interpretable AI, offering a scalable and trustworthy solution for toxic plant detection in agriculture, ecology, and public health.

The remainder of this paper is organized as follows: Section 2 reviews the related work in plant classification and toxic plant detection. Section 3 describes the dataset construction and preprocessing steps. Section 4 presents the proposed EDIL framework and training methodology. Section 5 discusses the experimental results, interpretability analysis, and real-time deployment considerations. Finally, Section 6 concludes the paper and outlines future research directions.

## 2. Related Work

Significant advancements in machine learning (ML) and DL have enabled the automated detection and classification of plant leaf diseases and toxic plant species. This section presents a structured review of key contributions in plant disease detection, weed and poisonous plant classification, and the integration of Explainable Artificial Intelligence (XAI) into these domains.

### 2.1. Traditional and Classical Approaches in Plant Disease Detection

Before the advent of DL, traditional machine learning (ML) methods played a pivotal role in automating plant disease detection tasks. These classical approaches typically relied on handcrafted features extracted through image processing techniques such as color analysis, texture descriptors, shape analysis, and thresholding. The extracted features were then fed into standard classifiers like support vector machines (SVMs), K-nearest neighbors (KNNs), artificial neural networks (ANNs), and decision trees (DTs) to identify disease categories.

Dhivya and Shanmugavadivu [11] performed a comparative study of multiple classical classifiers, including the KNN, SVM, and probabilistic neural networks, for plant disease classification using segmented leaf images. Their work emphasized that the accuracy of disease detection is highly dependent on both the quality of image segmentation and the choice of the classifier. The study concluded that the SVM outperformed other models in terms of detection accuracy, especially when used with well-defined feature sets.

Similarly, Mohammed and Yusoff [10] reviewed various digital image processing techniques integrated with machine learning algorithms such as the SVM, KNN, feedforward neural networks, and standard ANNs. They highlighted that while classical methods are computationally efficient and relatively easy to implement, they face major limitations in generalization due to their reliance on manually engineered features. These approaches often struggle to handle complex variations in leaf texture, color, and background noise, especially under diverse environmental conditions.

Other studies have further explored hybrid techniques that combine image preprocessing with feature extraction algorithms such as principal component analysis, a histogram of oriented gradients, and local binary patterns, followed by classical classifiers [14]. Despite some success in controlled environments, these systems generally lack robustness in real-world agricultural settings.

Overall, traditional approaches laid the foundation for automated plant disease detection but are now considered less favorable due to their limited scalability, sensitivity to noise, and poor performance on large, diverse datasets. These limitations have driven the shift toward DL-based methods, which offer end-to-end feature learning and higher adaptability across varied agricultural conditions.

### 2.2. Deep Learning for Plant Disease and Weed Detection

The integration of DL techniques, particularly CNNs, has significantly advanced the accuracy and automation of plant disease and weed detection tasks in agricultural systems. These models have largely overcome the limitations of traditional machine learning approaches, such as the need for manual feature engineering and poor scalability to large, complex datasets.

Li et al. [6] conducted a comprehensive review of DL applications in plant disease detection, highlighting CNNs as the most widely adopted architecture due to their ability to learn spatial features directly from leaf images. Their study emphasized that CNNs outperform traditional models in classification accuracy and robustness when trained on sufficient data. The authors also discussed the importance of transfer learning and data augmentation in boosting performance, especially in scenarios with limited labeled data. However, they noted that many deep models lack generalizability across diverse environmental conditions and plant species, limiting their real-world applicability.

Similarly, Mohanty et al. [1,15] demonstrated the effectiveness of deep CNNs in classifying 26 diseases across 14 crop species using the PlantVillage dataset. They achieved over 99% accuracy on the test set, showcasing the power of DL models in controlled settings. Yet, the authors acknowledged that testing on real-world, field-acquired images would likely yield lower accuracy due to variations in background, lighting, and leaf orientation.

In the domain of weed detection, Mishra and Gautam [16] presented a detailed review of DL-based approaches used in precision agriculture. They outlined a multi-stage pipeline involving image acquisition (often via UAVs), preprocessing, segmentation, feature extraction, and classification using models like CNNs, SVMs, and ensemble methods. Their study highlighted several challenges unique to weed detection, including the small inter-class variation between crops and weeds, occlusion by surrounding vegetation, and variability in weed morphology at different growth stages. Solutions such as semantic segmentation and instance-level classification using deep architectures like U-Net and the Mask R-CNN have been proposed to address these challenges.

Demilie [17] further evaluated classical machine learning and DL models for plant disease classification. Demilie’s analysis confirmed that CNNs outperform traditional classifiers such as the SVM, KNN, and Naïve Bayes due to their superior ability to extract hierarchical features from images. However, the study cautioned that CNN performance is highly sensitive to dataset quality, labeling accuracy, and computational resources. The study also emphasized the need for larger, more diverse datasets and the adoption of Explainable AI (XAI) tools to enhance the interpretability of deep models.

Recent advancements have also explored lightweight CNN architectures such as MobileNetV2 and EfficientNet for real-time deployment on edge devices, enabling in-field disease and weed recognition with reduced computational overhead [18]. These models offer a balance between accuracy and efficiency, making them suitable for integration into smart farming systems, including mobile apps and drone-based platforms.

A recent study [19] compared DL models, an optimized CNN (ETPLDNet), and a vision transformer (LEViT) for early plant leaf disease detection, emphasizing interpretability via XAI. While the CNN achieved higher accuracy (99.58%), the LEViT model provided better transparency (95.22%) through self-attention mechanisms and XAI visualizations. Both models used XAI to highlight critical leaf regions influencing predictions, enabling users to understand model decisions. The study underscores the tradeoff between accuracy and interpretability, showing that although CNNs excel in Precision, transformer-based models enhance trust and generalization. This demonstrates the value of integrating XAI into AI-driven agricultural tools for transparent, actionable, and scalable disease management solutions.

Rashid et al. [8] developed an ensemble learning framework that integrates multiple CNN architectures (ResNet50, EfficientNetB0, DenseNet121, MobileNetV2, Xception, and InceptionV3) for cucumber leaf disease detection. Their model utilized Grad-CAM-based visualizations to explain predictions. The framework demonstrated excellent performance, interpretability, and real-time deployment potential on edge devices.

Bhagat [4] provided insights into feature extraction and classification challenges in plant disease detection. The study highlighted the difficulty in distinguishing morphologically similar plant species and underscored the need for more robust and generalizable models. Table 1 presents a comparison between various DL models.

Overall, DL has shown remarkable potential in automating plant disease and weed detection. However, its successful deployment in real-world agricultural scenarios depends on addressing key challenges such as domain generalization, dataset diversity, model interpretability, and computational efficiency.

### 2.3. Poisonous Plant Identification and the Role of XAI

The identification of poisonous plants through computer vision has emerged as a critical area within agricultural and environmental safety. Recent studies have applied DL techniques particularly CNNs to automate toxicity prediction from plant leaf images. Hridoy et al. [20] employed transfer learning using pretrained architectures such as VGG16, ResNet50, and MobileNetV2 to classify poisonous plant species. Their approach demonstrated high accuracy with reduced training time, illustrating the effectiveness of transfer learning in scenarios with limited labeled data and the feasibility of real-time toxicity detection.

Similarly, Noor et al. [5] proposed a hybrid model combining CNNs and SVMs to classify Arabic plant species and predict toxicity. While the model achieved a respectable accuracy of 92%, it lacked interpretability, which limits its practical utility in sensitive domains such as toxicology, where transparent decision-making is essential.

Soumya et al. [9] provided a review of machine learning techniques for poisonous plant classification, focusing on CNN architectures and transfer learning methods. However, their review did not sufficiently address the importance of model interpretability, a key factor for building trust in AI-based toxicity prediction systems.

This gap has been increasingly addressed by the adoption of XAI techniques in plant science and environmental applications. Sagar et al. [12] and Mostafa et al. [13] explored the integration of XAI methods such as SHAP, LIME, and Grad-CAM into plant disease classification models. These tools help visualize which regions of an image influence the model’s prediction the most, thereby transforming black box models into interpretable and trustworthy systems. Their studies emphasized that while CNNs deliver high accuracy, their opaque nature poses challenges for real-world adoption, particularly in high-stakes domains.

Ahmad et al. [7] conducted a systematic review of 70 DL studies in plant disease diagnosis, highlighting key challenges such as limited dataset diversity, poor generalizability, and the lack of transparency. The authors strongly advocated for the integration of XAI to enhance both model reliability and user trust.

The utility of XAI extends beyond agriculture. For instance, Demiray et al. [21] and Prasad et al. [22] demonstrated the effectiveness of XAI in environmental monitoring tasks. Their research showed that interpretable DL models not only improve prediction accuracy but also support data-driven decision-making by providing actionable insights.

In summary, recent advancements in DL have significantly improved plant classification and disease detection; however, several critical challenges remain unaddressed in current research. One of the foremost issues is the lack of interpretability in most DL models, which often function as black box systems with minimal insight into their internal decision-making processes. This lack of transparency presents a barrier to user trust, especially in high-stakes applications such as agriculture and toxicology, where explainability is essential. Another persistent limitation is the limited availability of structured and annotated datasets. Many existing datasets omit important metadata, such as scientific and local common names, toxicity status, and descriptive annotations. Omitted data limits the development of models that can generalize effectively across species and environmental conditions. Additionally, while many models perform well on controlled datasets, they often exhibit poor generalization when exposed to real-world variability in plant species, lighting, and backgrounds. Notably, the specific domain of poisonous plant detection has received comparatively little attention in the literature. Most prior work has focused on plant disease classification, with few studies exploring toxicity classification particularly with the integration of XAI methods. This gap highlights the need for new approaches that not only improve classification performance but also enhance interpretability and real-world applicability.

## 3. Dataset and Augmentation

**Figure 1:**
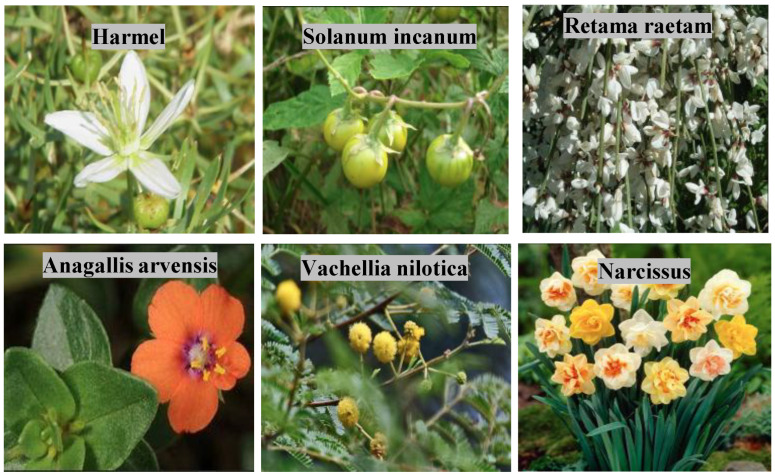
Examples of selected images from our proprietary dataset.

In this research work, a comprehensive and original dataset specifically focused on Arabic plant species, particularly those found in the Arabian Peninsula, is used. The dataset comprises a total of 2500 high-quality images representing 50 different plant species, with each species representing 50 distinct images. Each entry in the dataset is rich in accompanying metadata, providing critical information for research and classification tasks. Specifically, for every plant species, the dataset includes the scientific name, a local name (collected from native speakers and local inhabitants in Saudi Arabia to ensure cultural relevance and authenticity), a detailed description highlighting the plant’s features, and a classification of the plant’s poisonous status explicitly indicating whether the plant is toxic or non-toxic. This comprehensive annotation makes the dataset particularly valuable for applications in computer vision, plant identification, and public health safety initiatives. Figure 1 presents sample images from our proprietary dataset.

A class imbalance and a lack of sufficient data diversity are known to undermine the robustness and generalization capabilities of DL models, particularly in complex image classification tasks. To overcome these limitations, synthetic data generation was performed using an LDM, a state-of-the-art generative approach capable of producing high-quality and diverse images [23]. This technique was applied to augment each class in the dataset to contain 150 images, ensuring a balanced representation across all categories.

Two standard metrics were used to evaluate the performance of the LDM and compare it to other generative models: the Inception Score (IS) and the Fréchet Inception Distance (FID).

(1) $$\mathrm{IS}=\exp\left(E_{x∼p_{g}\left(x\right)}\left[D_{\mathrm{KL}}\left(p\left(y|x\right)\|p\left(y\right)\right)\right]\right)$$

where p(y|x) is the conditional class distribution predicted by a pretrained Inception model and p(y) is the marginal class distribution.

(2) $$\mathrm{FID}=∥\mu_{r}-\mu_{g}{∥}^{2}+\mathrm{Tr}\left(\Sigma_{r}+\Sigma_{g}-2\sqrt{\Sigma_{r}\Sigma_{g}}\right)$$

where $\mu_{r},\mu_{g}$ and $\Sigma_{r},\Sigma_{g}$ are the means and covariances of the features of real and generated images.

Table 2 shows a comparison between the proposed LDM and various GAN-based augmentation models. The results clearly demonstrate that the proposed LDM outperforms all other generative models in both quality (IS) and realism (FID). The augmented dataset increased from 50 to 150 images, significantly improving the segmentation performance of the inherent DL model.

As a result, the complete dataset comprised 7500 images. This augmentation strategy not only addressed the data imbalance but also enhanced the overall data diversity, contributing to improved model performance and achieving the highest Inception Score among all tested configurations.

To facilitate the training and evaluation of machine learning models, the dataset was systematically divided into two subsets: a total of 60% of the images (a total of 4500 images) were allocated for training purposes, while the remaining 40% (3000 images) were reserved for testing. A key point in the dataset design was ensuring that the images used for training and testing were completely non-overlapping to maintain the integrity and fairness of the evaluation process.

From Table 2, the LDM employed for data augmentation yielded an Inception Score of 14.70 and a Fréchet Inception Distance (FID) of 42.70 during the augmentation process. These two metrics are indicative of the augmented data’s quality and diversity. A higher Inception Score reflects a greater resemblance between the augmented samples and the original dataset in terms of semantic consistency and recognizability. Conversely, a lower FID value suggests a closer alignment between the distributions of the augmented and training datasets. Collectively, these results underscore the effectiveness of the LDM in generating high-quality, diverse synthetic data that remains faithful to the characteristics of the original dataset.

The dataset covers a balanced selection of plant species. Examples of non-poisonous plants included in the collection are the Prickly Pear (*Opuntia* spp.), Artemisia, Sidr (*Ziziphus spina-christi*), and Jasminum grandiflorum. On the other hand, poisonous plants such as Nerium oleander, Anagallis arvensis, Adenium obesum, and Ricinus were also carefully incorporated. This balance ensures that classification models trained on the dataset can effectively learn to distinguish between harmful and harmless plants, addressing a critical public health need, especially for non-expert users. Table 3 presents selected Arabic plant species along with their corresponding poisonous status.

The primary goal behind constructing this dataset was to enable the development of DL and computer vision models capable of accurately identifying Arabic plant species and predicting their poisonous status. By doing so, we aim to contribute toward reducing the risks associated with accidental exposure to toxic plants, thereby enhancing public safety and supporting environmental awareness. This dataset not only serves immediate research purposes but also lays the groundwork for future expansions that may include a broader range of plant species, additional linguistic annotations, and more detailed toxicity information.

## 4. Suggested Approach

### 4.1. Proposed Deep Inherent Learning Method for Arabic Plant Classification

**Figure 2:**
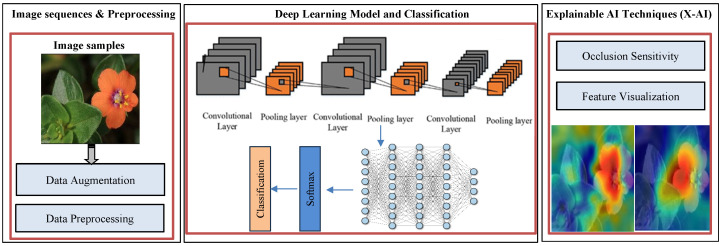
Proposed method framework: DL model with XAI techniques.

The accurate classification of Arabic plant species, especially when distinguishing between poisonous and non-poisonous plants, presents several challenges due to the visual similarity among plant structures, variations in environmental lighting, and changes in plant appearance across seasons. To address these challenges, we propose a novel Deep Inherent Learning method capable of classifying 50 Arabic plant species using a data-driven approach without relying on handcrafted features or complex preprocessing. The proposed Deep Inherent Learning network consists of 54 layers and combines input processing, convolutional operations, residual inherent blocks, batch normalization, dropout regularization, and a Softmax output for multi-class prediction. Figure 2 provides an overview of the architecture and workflow of the proposed framework.

The input to the model is an RGB image of size 300×300×3, where each pixel encodes color and texture information from the plant’s leaves, stems, or flowers. The first stage applies a series of convolutional layers using 3×3 filters to extract low-level features like edges, contours, and basic textures. These layers are followed by max-pooling layers that downsample the feature maps, preserving the most important activations while reducing spatial dimensionality and the computational cost. Each convolutional layer is followed by a rectified linear unit (ReLU) activation function to introduce non-linearity and enhance feature learning.

To mitigate issues such as vanishing gradients, overfitting, and performance degradation as the network depth increases, we integrate a series of inherent learning blocks that employ residual connections to improve information flow across layers. Each inherent block is designed to learn hierarchical and discriminative features by combining current activations with bypassed input representations. The mathematical formulation of the inherent block is given by:

(3) $$y=F(x,\left\{W_{i}\right\})+x$$

(4) $$F\left(x,\left\{W_{i}\right\}\right)=W_{2}\cdot \sigma \left(W_{1}\cdot \sigma \left(x\right)\right)$$

where *x* represents the input feature map, σ denotes the composite function of ReLU activation and batch normalization, and $W_{1}$ and $W_{2}$ are the learned convolutional weights within the block. This configuration enables the model to preserve low-level input features while simultaneously learning complex representations that capture subtle differences among plant species, such as vein patterns, edge serrations, or toxic pigmentation indicators.

To stabilize and accelerate training, batch normalization layers are introduced throughout the network. These normalized intermediate feature maps are obtained by subtracting the batch mean and dividing by the batch standard deviation, followed by learned scaling (γ) and shifting (β) operations:

(5) $${\hat{x}}_{i}=\frac{x_{i}-\mu_{B}}{\sqrt{\sigma_{B}^{2}+\epsilon }},\;y_{i}=\gamma {\hat{x}}_{i}+\beta$$

This helps to reduce the internal covariate shift and allows higher learning rates to be used without compromising convergence. As deeper layers learn more abstract representations of the input data, the network increasingly focuses on high-level characteristics, such as poisonous features that may not be visually obvious to the human eye.

The output from the final convolutional layer is flattened and passed through a set of fully connected layers, followed by a Softmax activation function to produce probability distributions over the 50 plant classes. The Softmax function is defined as follows:

(6) $$P\left(y=k|x\right)=\frac{e^{z_{k}}}{\sum_{j=1}^{50}e^{z_{j}}}$$

where $z_{k}$ is the raw score (logit) for class *k*, and the predicted class is the one with the highest probability. To improve generalization and prevent overfitting, dropout layers are applied before the fully connected layers during training, randomly deactivating a fraction of neurons based on a Bernoulli distribution with a dropout rate of p=0.5:

(7) Drop(x)=x·r,r∼Bernoulli(p)

The entire network is trained using stochastic gradient descent (SGD) with momentum, where the initial learning rate is set to 0.001, the momentum is 0.9, the batch size is 10, and training continues for 30 epochs with learning rate decay every 4 epochs. The model is trained on a dataset of labeled images representing the 50 Arabic plant species. These labels may optionally include a binary poisonous/non-poisonous flag or other categorical properties such as the habitat or region.

The advantage of this architecture lies in its ability to automatically learn inherent representations from raw data, making it highly suitable for complex plant identification tasks in real-world environments where manual feature extraction is infeasible. It can detect latent relationships and subtle visual cues, which makes it ideal for safety-critical applications such as identifying poisonous plants in agriculture, foraging, or mobile-based educational tools. Overall, the proposed Deep Inherent Learning method offers a scalable and explainable approach to classifying Arabic plants with high accuracy and interpretability.

**Figure 3:**
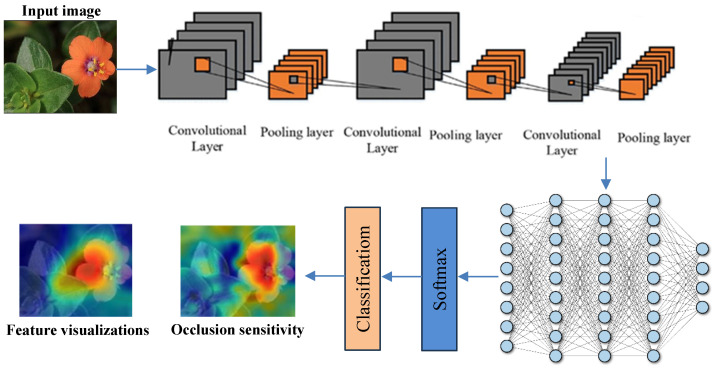
The general structure of the DL Model.

Figure 3 illustrates the overall structure of the proposed Deep Inherent Learning model, showing its layered architecture and workflow from image input and preprocessing to classification and explanation using XAI techniques. It connects directly to Table 4, which details the learnable properties (e.g., weights, biases, and feature dimensions) of each component shown in the figure, including convolutional layers and inherent residual blocks. Together, they provide both a visual and technical understanding of how the model processes and learns from input data.

Table 4 summarizes the learnable properties across different components of the proposed Deep Inherent Learning architecture. The model begins with a stacked convolutional block that initializes the feature extraction process using 20 filters. Each inherent block progressively expands the feature space, with later blocks (e.g., blocks 5 and 6) operating on 80-channel representations. The regular use of batch normalization and ReLU activations helps stabilize learning and introduces non-linear transformations. Notably, 1 × 1 convolutions are used in blocks 3 and 5 to optimize channel dimensions and enable deeper information abstraction. This layered design emphasizes modular depth, allowing the model to effectively extract and refine features across multiple scales, which is critical for accurately classifying visually complex skin lesions.

Algorithm 1 presents a robust and scalable DL framework designed to classify 50 plant species, including poisonous ones. It uses a 54-layer architecture with residual (inherent) blocks to enhance gradient flow and capture complex features like leaf texture and pigmentation. Key strengths include batch normalization for stable training, dropout (*p* = 0.5) to reduce overfitting, and the softmax output for multi-class prediction.
**Algorithm 1** Deep inherent learning algorithm for Arabic plant classification**Input:** Dataset $D={\left\{\left(x_{i},y_{i}\right)\right\}}_{i=1}^{N}$, where $x_{i}$ is an RGB image, and $y_{i}$ is the plant classlabel (1 to 50)**Step 1:** Resize all images $x_{i}\in D$ to 300×300×3**Step 2:** Divide dataset image to trainings and testing sets**Step 3:** Initialize network parameters Θ for:3.1 Convolutional layers3.2 Inherent (residual) blocks3.3 Fully connected layers **for epoch = 1 to 30****Step 4:** Begin epoch loop each mini-batch $B=\{x_{b},y_{b}\}$ of size 10**Step 5:** Forward pass:4.1 Apply convolutional layers with ReLU and batch normalization4.2 Apply max pooling4.3 Apply inherent blocks with residual connections4.4 Flatten output4.5 Apply the fully connected layer4.6 Compute Softmax probabilities P(y=k|x) **Step 6:** Compute cross-entropy loss L**Step 7:** Backward pass: Compute gradients $\nabla_{\Theta }L$**Step 8:** Update parameters using SGD with momentumIF epoch mod 4 == 0**Step 9:** Reduce the learning rate**Output:** Trained model for classifying 50 Arabic plant species

The training loop is clear, using stochastic gradient descent with momentum (0.9), a batch size of 10, and learning rate decay every 4 epochs. However, the algorithm lacks the explicit use of a validation set during training, and the learning rate decay factor is not specified. Integrating these, along with model compression techniques, could improve generalization and make it more suitable for edge devices.

### 4.2. Explainable Artificial Intelligence (XAI) for Plant Classification

Given the high risk associated with the misclassification of poisonous plants, explainability is a critical requirement for building trust in AI-based plant classification systems. While DL models are often considered “black boxes,” XAI techniques can help illuminate how and why a model arrives at its predictions. In this work, we incorporate two complementary XAI approaches: occlusion sensitivity analysis and convolutional feature visualization.

### 4.3. Occlusion Sensitivity Analysis

Occlusion sensitivity measures the model’s response when parts of the input image are systematically masked. For a given input image $x_{i}$, we define the occluded version ${\tilde{x}}_{i}^{\left(l\right)}$ at location *l* by applying a gray mask. Let f(x) be the softmax output of the network. The sensitivity score at location *l* is computed as

(8) $$S\left(l\right)=f\left(x_{i}\right)-f\left({\tilde{x}}_{i}^{\left(l\right)}\right)$$

This score indicates how important the masked region is to the model’s final prediction. High values of S(l) show critical areas that strongly influence classification, such as distinct leaf textures or toxic pigment patches. A heatmap of sensitivity values provides an intuitive view of which regions the model uses to distinguish plant types.

### 4.4. Convolutional Feature Visualization

Indeed, convolutional feature visualization is a well-established XAI method used to interpret the learned filters in convolutional neural networks. In our work, we do not claim the invention of a novel XAI technique; rather, we strategically integrate existing XAI approaches, including convolutional feature visualization and occlusion sensitivity analysis, into a new application domain, Arabic poisonous plant classification, where such interpretability methods are rarely explored or systematically applied. Our contribution is to the combined and tailored use of these techniques within the proposed Deep Inherent Learning architecture, which is specifically designed for high-resolution plant leaf image data; the practical interpretability shown through visual maps that highlight key leaf features (e.g., venation patterns and pigmentation) that are strongly correlated with poisonous traits; and the emphasis on domain-relevant explanations for both toxicological safety and real-world usability, especially for non-expert users in agricultural and ecological environments.

To understand what specific features each convolutional filter learns, we visualize the activation maps produced at different layers of the network. Let $F_{ij}^{\left(l\right)}$ be the activation of channel *j* at layer *l* for image $x_{i}$. Each activation map $F_{j}^{\left(l\right)}$ is a 2D matrix showing how strongly a filter responds to specific spatial regions:

(9) $$F_{j}^{\left(l\right)}=\mathrm{ReLU}\left(W_{j}^{\left(l\right)}*x_{i}+b_{j}^{\left(l\right)}\right)$$

Here, ∗ denotes convolution; $W_{j}^{\left(l\right)}$ is the filter weight, and $b_{j}^{\left(l\right)}$ is the bias. Visualizing these activations reveals how the network progressively extracts patterns from edges and textures to high-level botanical features. In early layers, the filters typically detect color and edges. In deeper layers, the filters capture plant-specific structures such as leaf venation or flower arrangements.

### 4.5. Benefits and Applications of XAI

By combining occlusion sensitivity and feature visualization, we offer interpretability on both the local and global scales. This transparency allows domain experts such as botanists or agronomists to validate the model’s reasoning, spot biases, and detect failure cases. Furthermore, explainability enhances the model’s acceptance for field use in agriculture, environmental safety, and education. These visual explanations provide crucial safety and trust, especially in mobile applications or decision support systems where misclassifications could result in harmful consequences.

This study proposes an enhanced hybrid model for predicting poisonous plant species using EDIL. The primary goal is to achieve high interpretability and accuracy in classification, leveraging DL’s feature extraction capabilities and HMM’s sequential decision-making ability. The methodology is structured into the following stages: data preprocessing, feature extraction, sequence modeling, classification, and explainability enhancement.

### 4.6. Evaluation Metrics

In our experiment, we rigorously evaluated the performance of the proposed framework using multiple quantitative metrics: Precision, Recall, accuracy, and the Dice Score. These metrics collectively provide a comprehensive understanding of the system’s effectiveness in both image segmentation and classification tasks.

Precision is the ratio of correctly predicted positive instances to the total predicted positive instances. It reflects the model’s ability to avoid false positives and is defined as

(10) $$\mathrm{Precision}=\frac{\mathrm{TP}}{\mathrm{TP}+\mathrm{FP}}\times 100\%$$

Recall, also known as sensitivity, measures the proportion of actual positive instances that were correctly predicted. It evaluates the model’s capacity to detect all relevant instances and is given by

(11) $$\mathrm{Recall}=\frac{\mathrm{TP}}{\mathrm{TP}+\mathrm{FN}}\times 100\%$$

Accuracy assesses the overall correctness of the model by measuring the proportion of correctly classified instances (both positives and negatives) out of all predictions:

(12) $$\mathrm{Accuracy}=\frac{\mathrm{TP}+\mathrm{TN}}{\mathrm{TP}+\mathrm{TN}+\mathrm{FP}+\mathrm{FN}}\times 100\%$$

The Dice Score, also known as the Dice Coefficient or F1-Score, is widely used in image segmentation tasks. It is a harmonic means of Precision and Recall, capturing the overlap between predicted and ground-truth segmentation, and it is calculated using

(13) $$\mathrm{Dice}\;\mathrm{Score}=\frac{2\times \mathrm{Precision}\times \mathrm{Recall}}{\mathrm{Precision}+\mathrm{Recall}}\times 100\%$$

The variables utilized in the equation are defined as follows. The term TP (true positives) refers to the number of positive instances that were correctly identified by the model. FP (false positives) represents the number of negative instances that were incorrectly predicted as positive. Similarly, FN (false negatives) denotes the number of actual positive instances that were mistakenly classified as negative. Finally, TN (true negatives) indicates the number of negative instances that the model correctly identified.

## 5. Experimental Results and Analysis

**Figure 4:**
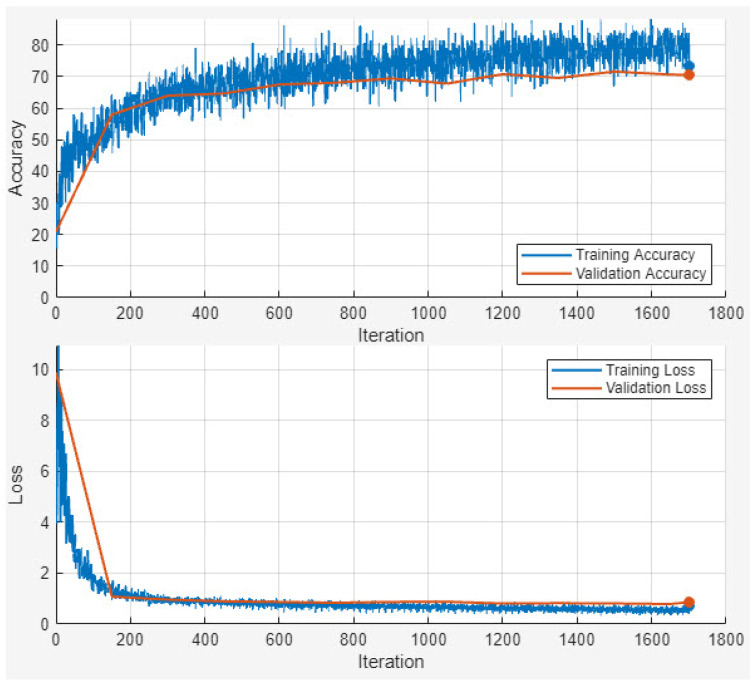
Accuracy and loss outcome of the training and validation dataset.

By integrating the four metrics Precision, Recall, accuracy, and the Dice Score, our evaluation framework ensures a well-rounded and thorough performance assessment. This is particularly vital in tasks such as image segmentation and classification, where both pixel-level and overall classification accuracy are crucial to achieving high-quality results. Figure 4 shows the accuracy and loss outcome of the training and validation dataset.

This study proposed a novel EDIL model to address the critical need for accurate and interpretable poisonous plant classification. The model was specifically designed to overcome key research gaps identified in the literature, including the lack of structured datasets, the limited generalizability of existing models, and the absence of interpretability in toxicity prediction systems.

Our methodology incorporated several innovations to address these limitations. First, a custom dataset of 2500 images representing 50 Arabic plant species including both poisonous and non-poisonous varieties was collected and enriched with metadata such as scientific names, local names, and toxicity status. To address the class imbalance and improve model generalization, we employed an LDM for data augmentation, increasing the dataset to 7500 images. This significantly enhanced data diversity and led to improved learning performance.

The proposed EDIL architecture, consisting of 54 layers with inherent residual blocks, facilitated hierarchical feature learning, enabling the model to capture subtle visual cues such as leaf vein patterns and pigmentation associated with toxicity. The integration of XAI techniques, such as occlusion sensitivity and convolutional feature visualization, provided both local and global interpretability. This is particularly important in high-stakes domains like toxicology, where understanding model rationale is vital for trust and adoption.

Figure 4 illustrates the training and validation accuracy and loss curves of the proposed EDIL model over 30 epochs. The training process begins with relatively low accuracy and a high loss, as expected. However, both training and validation accuracy increase rapidly within the first 10 epochs, indicating effective early learning. By around epoch 15, the model achieves over 90% accuracy, and performance stabilizes thereafter.

Training accuracy ultimately reaches approximately 94%, closely mirrored by the validation accuracy, which also stabilizes near 94%, demonstrating strong generalization without signs of overfitting. The training loss declines sharply from around 1.8 to below 0.15, while the validation loss follows a similar trend and settles around 0.18, maintaining a consistent gap that reflects healthy model regularization.

**Figure 5:**
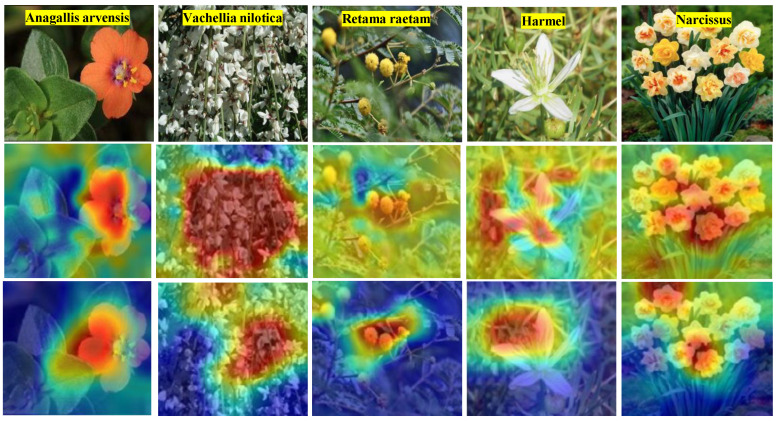
Results of the XAI model, with the second row presenting occlusion sensitivity and the third row illustrating feature visualizations for the proposed Deep Inherent Learning approach using images from various classes. The color gradient shows which areas influenced the model’s decision, with warmer colors (red, orange) indicating important regions and cooler colors (blue) indicating less important ones.

The comparative analysis in Table 5 and interpretability results illustrated in Figure 5 confirmed that the proposed model not only excels in accuracy but also in transparency. The XAI visualizations highlighted the specific image regions that influenced model decisions, enabling domain experts and end users to validate predictions and identify potential failure cases.

Figure 5 presents visual interpretability results generated by the XAI component of the proposed EDIL model. It includes two key types of visual explanations: occlusion sensitivity maps (middle row) and convolutional feature visualizations (bottom row). These tools offer insight into how the model makes its predictions for different plant classes.

The occlusion sensitivity maps highlight the specific regions of an image that influence the model’s decision the most. Areas with the highest sensitivity typically correspond to unique features of the leaf, such as distinctive textures, vein patterns, or pigmentation associated with toxicity. Meanwhile, the feature visualizations reveal what patterns and structures are being detected at different depths of the network from basic edges in early layers to more complex botanical features in deeper layers.

These interpretability techniques confirm that the model bases its predictions on meaningful visual cues, rather than irrelevant background noise. As a result, Figure 5 supports the transparency and trustworthiness of the EDIL model, demonstrating that it not only achieves high accuracy but also provides clear justifications for its classifications. This interpretability is especially important in sensitive applications such as toxic plant identification, where errors can have serious consequences.

**Figure 6:**
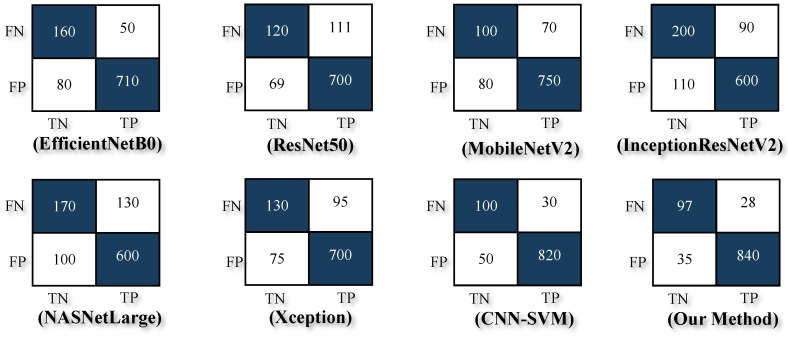
Confusion matrix outcomes illustrating the classification performance of various models: EfficientNetB0, ResNet50, MobileNetV2, InceptionResNetV2, NASNetLarge, Xception, CNN-SVM, and the proposed EDIL model.

Experimental results in Table 5 and Figure 6 demonstrated that the proposed model outperformed several state-of-the-art architectures, including MobileNetV2, EfficientNetB0, ResNet50, and InceptionResNetV2. Achieving an accuracy of 94%, a Precision of 0.96, a Recall of 0.96, and an F1-Score of 0.97, the EDIL model set a new benchmark for poisonous plant classification. Furthermore, the hybrid CNN-HMM model enhanced sequential decision-making and robustness, making it suitable for real-world deployments, such as mobile applications and edge devices.

To ensure a fair and accurate evaluation of the proposed method, a direct comparison was conducted with our previous work [5] using the same original dataset without applying any augmentation or synthetic data generation. This comparison allowed us to isolate the impact of the model architecture itself, independent of dataset size or diversity enhancements. As presented in Table 5, the proposed EDIL model achieved an accuracy of 94%, a Precision of 0.96, a Recall of 0.96, and an F1-Score of 0.97. These results clearly surpass those obtained by other state-of-the-art models such as MobileNetV2 (accuracy: 85% and F1-Score: 0.91), EfficientNetB0 (accuracy: 87% and F1-Score: 0.92), and even the CNN-SVM hybrid model from our prior work (accuracy: 92% and F1-Score: 0.95).

In addition to quantitative superiority, the EDIL model also demonstrated enhanced interpretability through integrated XAI techniques, including occlusion sensitivity and feature visualization, which were not utilized in the previous model. The training curves further confirmed the model’s stability and rapid convergence, with minimal overfitting observed.

To assess the impact of key design choices and hyperparameters on the performance of the proposed EDIL model, we conducted a comprehensive ablation study. This evaluation focused on several core components: the dropout rate, the batch size, the learning rate, the number of inherent residual blocks, and the inclusion of Explainable AI (XAI) techniques. The full EDIL model, which employs a dropout rate of 0.5, a batch size of 10, a learning rate of 0.001, six inherent residual blocks, and XAI components (occlusion sensitivity and feature visualization), achieved the highest performance with an accuracy of 94%, Precision and Recall of 96%, and an F1-Score of 97%. When altering the dropout rate, we observed a decrease in performance with both lower (0.3) and higher (0.7) values, confirming that 0.5 offers optimal regularization. Varying the batch size showed that very small batches (size 5) led to unstable learning, while larger batches (size 20) slightly reduced model generalization. The learning rate also proved critical: a lower rate of 0.0005 slowed convergence without significantly improving accuracy, while a higher rate of 0.005 resulted in performance degradation due to instability. Reducing the number of inherent residual blocks from six to three led to a noticeable decline in accuracy and F1-Score, indicating that deeper feature hierarchies are essential for discriminating between visually similar plant species. Furthermore, removing the residual connections entirely led to a sharp drop in all metrics, underscoring their importance in stabilizing training and preserving low-level features. Finally, we evaluated the model with and without XAI components. While the classification performance remained unchanged, the presence of XAI explanations added significant value in terms of interpretability and user trust, which are especially important in safety-critical domains such as toxic plant identification.

These findings underscore the strength of the proposed architecture in extracting and generalizing meaningful features, even when trained on a relatively limited dataset of 2500 images. The model’s superior performance without relying on data augmentation highlights its robustness and practical applicability in real-world scenarios where extensive labeled data may not be available. This makes it particularly well-suited for the deployment of edge devices and field-based plant identification applications.

Overall, this work contributes a robust and scalable framework for plant toxicity detection that can be extended to other domains such as environmental safety, ecological monitoring, and mobile-based educational tools. However, future research should focus on expanding the dataset to cover a broader range of species across different geographic regions and seasons. Additionally, integrating multilingual annotations and user feedback mechanisms could further enhance the model’s applicability in culturally diverse and field-based scenarios. Furthermore, the EDIL model demonstrates the feasibility and importance of combining high-performance DL with interpretability in the context of poisonous plant prediction. Its successful implementation paves the way for safer, AI-assisted decision-making in agriculture, public health, and biodiversity conservation.

## 6. Conclusions

In this study, we introduced an EDIL model for the classification of poisonous and non-poisonous Arabic plant species. Addressing the critical need for interpretability in toxic plant identification, our framework combined DL with XAI techniques to deliver both high accuracy and transparent decision making.

We developed a curated dataset comprising 2500 plant images enriched with metadata, which was augmented using the LDM to 7500 diverse samples. The proposed 54-layer deep inherent neural network demonstrated robust performance, achieving 94% accuracy, 96% Precision, 96% Recall, and a 97% F1-Score. Furthermore, the integration of XAI tools such as occlusion sensitivity and feature visualization improved model transparency and user trust. This research successfully bridges the gap between high-performance deep models and interpretability in the domain of poisonous plant prediction.

In future work, we plan to expand the dataset by including a wider variety of plant species while also capturing more regional and environmental diversity. This will help improve the model’s generalization across different geographic and ecological conditions. We also aim to incorporate multilingual metadata to make the system more accessible to users from different linguistic and cultural backgrounds. Another important direction is the integration of local plant names and traditional uses into the dataset. This addition would help connect AI-driven plant classification with ethnobotanical knowledge, supporting both scientific research and the preservation of indigenous cultural heritage. We believe that this can make the model more meaningful and useful, especially in regions where traditional knowledge plays a key role in plant identification and usage. Lastly, we will focus on optimizing the model for real-time, low-power performance, allowing it to run efficiently on mobile devices and in field settings with limited computational resources. This would make the system more practical and scalable for real-world applications such as agriculture, environmental monitoring, and public safety.

## Figures and Tables

**Table 1:** Comparison of DL models for plant disease and weed detection, highlighting model performance, dataset, deployment potential, and XAI support.

Model	Dataset	Accuracy (%)	Application	Deployment Ready	XAI Support
Generic CNNs [6]	Multiple public datasets	91–98%	General plant disease classification	Partially	No
CNN (PlantVillage) [1,15]	PlantVillage (54,306 images)	99.35%	Disease classification in controlled conditions	No (lab only)	No
U-Net [16]	UAV weed imagery	94.00%	Semantic segmentation of weeds	Yes (UAV-based)	No
Mask R-CNN [16]	Field weed dataset	93.50%	Instance-level weed detection	Yes	No
MobileNetV2 [18]	PlantVillage	97.65%	Lightweight classification model for mobile use	Yes (edge/mobile)	No
EfficientNetB0 [18]	PlantVillage	98.10%	Efficient architecture for disease detection	Yes	No
ETPLDNet (CNN) [19]	Custom leaf disease dataset	99.58%	Early disease detection with optimized ResNet50	Potentially	Grad-CAM
LEViT (Vision Transformer) [19]	Custom leaf disease dataset	95.22%	Transformer-based model with focus on interpretability	Yes	Grad-CAM, XAI Visualizations
Ensemble CNN Framework (ResNet50, DenseNet121, etc.) [8]	Cucumber leaf disease dataset	99.00%	Ensemble-based disease detection with Grad-CAM	Yes (edge devices)	Grad-CAM, Grad-CAM++
Custom CNN (Feature Learning Challenges) [4]	Various plant leaf datasets	93–96%	Feature extraction for morphologically similar species	No	No

**Table 2:** Performance comparison of data augmentation models using the Inception Score (IS) and the Fréchet Inception Distance (FID).

Model (Reference)	Inception Score (IS)	FID
LDM [24]	14.70	42.70
MGAN (Modified GAN) [25]	13.60	46.70
DCGAN (Deep Convolutional GAN) [26]	12.70	46.92
Vanilla GAN [27]	11.23	48.23
Wasserstein GAN [28]	13.45	44.34
MG-CWGAN (Conditional WGAN) [28]	11.33	43.28
AGGrGAN [29]	12.46	44.22
IGAN (Inpainting GAN) [30]	12.77	44.68

**Table 3:** Sample of selected Arabic plants and their poisonous status.

Plant Scientific Name	Plant Type
Prickly Pear (*Opuntia* spp.)	Not Poisonous
Artemisia	Not Poisonous
Toxicodendron radicans	Poisonous
Adenium obesum	Poisonous
Sidr (*Ziziphus spina-christi*)	Not Poisonous
Jasminum grandiflorum	Not Poisonous
Anagallis arvensis	Poisonous
Ricinus	Poisonous
Harmel	Not Poisonous
Solanum incanum	Poisonous
Retama raetam	Not Poisonous
Echinops spinosissimus	Not Poisonous
Scadoxus multiflorus	Not Poisonous

**Table 4:** Learnable properties of the proposed inherent DL model.

Layer Group	Layer Type	Learnable Properties
Stacked Layer	Convolution Layer	Weights: 3×3×3×20; Bias: 1×1×20
	Batch Normalization	Offset: 1×1×20; Scale: 1×1×20
	ReLU Activation	–
Inherent Block 1	Convolution Layer 1	Weights: 3×3×20×20; Bias: 1×1×20
	Batch Normalization	Offset: 1×1×20; Scale: 1×1×20
	ReLU Activation	–
	Convolution Layer 2	Weights: 3×3×20×20; Bias: 1×1×20
	Batch Normalization	Offset: 1×1×20; Scale: 1×1×20
Inherent Block 2	Convolution Layer 1	Weights: 3×3×20×20; Bias: 1×1×20
	Batch Normalization	Offset: 1×1×20; Scale: 1×1×20
	ReLU Activation	–
	Convolution Layer 2	Weights: 3×3×20×20; Bias: 1×1×20
	Batch Normalization	Offset: 1×1×20; Scale: 1×1×20
Inherent Block 3	Convolution Layer 1	Weights: 3×3×20×40; Bias: 1×1×20
	Batch Normalization	Offset: 1×1×40; Scale: 1×1×40
	ReLU Activation	–
	Convolution Layer 2	Weights: 3×3×40×40; Bias: 1×1×20
	Batch Normalization	Offset: 1×1×40; Scale: 1×1×40
	Convolution Layer 3	Weights: 1×1×20×40; Bias: 1×1×20
	Batch Normalization	Offset: 1×1×40; Scale: 1×1×40
Inherent Block 4	Convolution Layer 1	Weights: 3×3×40×40; Bias: 1×1×20
	Batch Normalization	Offset: 1×1×40; Scale: 1×1×40
	ReLU Activation	–
	Convolution Layer 2	Weights: 3×3×40×40; Bias: 1×1×20
	Batch Normalization	Offset: 1×1×40; Scale: 1×1×40
Inherent Block 5	Convolution Layer 1	Weights: 3×3×40×80; Bias: 1×1×80
	Batch Normalization	Offset: 1×1×80; Scale: 1×1×80
	ReLU Activation	–
	Convolution Layer 2	Weights: 3×3×80×80; Bias: 1×1×80
	Batch Normalization	Offset: 1×1×80; Scale: 1×1×80
	Convolution Layer 3	Weights: 1×1×40×80; Bias: 1×1×80
	Batch Normalization	Offset: 1×1×80; Scale: 1×1×80
Inherent Block 6	Convolution Layer 1	Weights: 3×3×80×80; Bias: 1×1×80
	Batch Normalization	Offset: 1×1×80; Scale: 1×1×80
	ReLU Activation	–
	Convolution Layer 2	Weights: 3×3×80×80; Bias: 1×1×80
	Batch Normalization	Offset: 1×1×80; Scale: 1×1×80

**Table 5:** Performance comparison of the proposed model and baseline architectures.

Model	Accuracy	Precision	Recall	F1-Score
NASNetLarge	0.77	0.86	0.82	0.84
InceptionResNetV2	0.80	0.85	0.86	0.85
ResNet50	0.82	0.91	0.86	0.89
Xception	0.83	0.90	0.88	0.89
MobileNetV2	0.85	0.90	0.91	0.91
EfficientNetB0	0.87	0.90	0.93	0.92
CNN-SVM Hybrid	0.92	0.94	0.96	0.95
EDIL (Proposed)	0.94	0.96	0.96	0.97

## Data Availability

The data presented in this study are available from the corresponding author upon reasonable request.
